# Bowel Function Score in Long-Term Follow-Up for Children with Hirschsprung Disease: OASIS-Holistic Care in Hirschsprung Disease Network Position Paper

**DOI:** 10.3390/children11111284

**Published:** 2024-10-24

**Authors:** Judith Lindert, Anders Telle Hoel, Eberhard Schmiedeke, Joe I. Curry, Stavros Loukogeorgakis, Eva Amerstorfer

**Affiliations:** 1 Department of Paediatric Surgery, University Hospital Rostock, 18057 Rostock, Germany; 2 Specialist Colorectal Service, Department of Specialist Neonatal and Paediatric Surgery, Great Ormond Street Hospital, London WC1N 3BH, UK; joe.curry@gosh.nhs.uk (J.I.C.); stavros.loukogeorgakis@gosh.nhs.uk (S.L.); 3 Department of Pediatric Surgery, Oslo University Hospital, 0372 Oslo, Norway; andho@ous-hf.no; 4 Clinic for Paediatric Surgery and Paediatric Urology, Klinikum Bremen Mitte, 28205 Bremen, Germany; eberhard.schmiedeke@klinikum-bremen-mitte.de; 5 UCL-GOSH Institute of Child Health, University College London, London WC1E 6BT, UK; 6 Department for Pediatric and Adolescent Surgery, Medical University of Graz, 8036 Graz, Austria; eva.amerstorfer@medunigraz.at

**Keywords:** Hirschsprung disease, continence score, scoring postoperative results, incontinence, bowel function, patient-reported outcome measurements (PROM)

## Abstract

Introduction: The assessment of bowel function in patients with Hirschsprung disease (HD) remains controversial, as several different bowel function scores are used in the literature and are therefore not suitable for reliable comparison. Conference Section: The OASIS-Holistic Care in Hirschsprung Disease Network Group addressed this issue and evaluated the most commonly used scores for their utility in HD patients. Scoping Review and Expert Group Consensus were performed. It was agreed that the ideal score for patients with Hirschsprung disease should be a comprehensive, validated score that allows reliable assessment of bowel function and continence, depicts changes according to management and natural history, and quantifies baseline parameters. This score should also enable comparisons of bowel function in patients born with HD worldwide. Concluding Remarks: The OASIS-Holistic Care in Hirschsprung Disease Network Group agreed that this ideal score does not yet exist and is therefore a future goal of the group. Meanwhile, clinicians should use a score for comparable, standardized, objective assessment, and the Rintala Score is suggested. Future developments may also lead to easily accessible patient-reported outcome measures and semi-automated dashboards that allow automated data extraction from electronic health records.

## 1. Introduction

Hirschsprung disease (HD) affects approximately 1 in 5000 live births, of whom 80–85% have HD confined to the rectosigmoid and 20–25% of patients have HD extending upwards to varying lengths [1]. HD is characterized by the absence of enteric ganglion cells in the affected bowel, resulting in chronic functional bowel obstruction. Most children are diagnosed with HD within their first year of life [1]. Corrective surgical strategies aim to resect the abnormally innervated bowel and connect the healthy, adequately innervated bowel to the distal rectum without leaving the affected bowel in situ [1,2,3].

Despite technically sound reconstruction in experienced hands, long-term functional impairment is common after pull-through surgery [3,4,5,6]. Bowel function can deteriorate significantly at any age, and longitudinal monitoring is recommended. Impaired bowel function is known to alter psychosocial functioning and quality of life (QoL), announcing the need for dedicated bowel management to mitigate these sequelae and enable children to be continent [7,8,9]. Therefore, highly specialized follow-up is required, and eventually, transition to adult services is recommended [1,10,11].

Besides increasing awareness of the need for a long-term follow-up of HD, there is no agreement on a standardized way of evaluating bowel function in the long-term follow-up, yet. The abundance of different classifications and scoring systems creates challenges in comparing the functional results in the literature [12].

A total of 84 different questionnaires exist to assess bowel function in colorectal diseases. Only 8 of them are validated, 15 are established and broadly used, and 61 are self-designed [13]. These questionnaires are mostly developed and mainly intended to be used in patients with an anorectal malformation (ARM) [12].

Although HD and ARM are two different entities with different reconstructive surgical procedures, these two pediatric colorectal conditions are usually combined in analyses of bowel function utilizing continence scores and bowel function outcome measurements [12].

Interestingly, the ERNICA (European Reference Network for rare Inherited and Congenital (digestive and gastrointestinal) Anomalies) guidelines on rectosigmoid HD do not discuss the use of bowel function scores nor does it recommend a certain score that should be used to assess bowel function [1].

There is currently no consensus on the most effective way to evaluate bowel function in long-term follow-up. Multiple bowel function scoring systems exist, yet their applicability and consistency in HD are varied. The purpose of this article is to provide an overview of appropriate tools for monitoring bowel function in patients with HD and to highlight the holistic, patient-centered care practices advocated by the OASIS-Holistic Care in Hirschsprung Disease Network Group. 

## 2. Conference Section

The OASIS-Holistic Care in Hirschsprung Disease Network Group prepared a session dedicated to “Continence Scores” to discuss this topic at the international “OASIS-Holistic Care in Hirschsprung Disease Symposium” in Warnemünde, Germany, in July 2023.

The working group “Continence Score of the OASIS-Holistic Care in Hirschsprung Disease Network Group” prepared the content of the session and discussion, which is reflected in this manuscript. The information was obtained through a scoping review of bowel function scores and other colorectal scores, which was presented and discussed in the session dedicated to Continence Scores. This session also included and further evaluated the perspectives of patient associations and affected families. A total of 98 participants from 11 countries shared experiences and discussed holistic aspects of the care of Hirschsprung disease. Attendees came from Germany, Netherlands, Poland, Italy, United Kingdom, Austria, Spain, Sweden, Norway, Switzerland, and France. A total of 85 pediatric surgeons, 8 representatives from patient associations, and 15 researchers (some of them clinicians as well) were present. A model for real-time data collection and assessment of standardized data outcomes using a pediatric bowel function and patient quality of life dashboard was presented [14]. Finally, overall considerations for achieving consensus among pediatric colorectal surgeons were discussed. The scores were discussed, and the opinions of patient representatives and patients were heard. A voting system was used, and when the majority agreed, the view was accepted for this statement.

### 2.1. Overview Bowel Function Assessment Tools Used in Hirschsprung Disease

The OASIS-Holistic Care in Hirschsprung Disease Network Group evaluated the following 8 bowel function scores during the Continence Score session for the applicability in HD during the Network meeting (Table 1). The following 8 scores were selected by the expert group preparing session after a review of the available scores.

### 2.2. Criteria for Bowel Function Assessment Tools Applicable in Patients with HD

A bowel function assessment tool for HD needs to address the following items:

- *Quantify baseline variables*
  Objective quantification may allow different team members to picture the situation and share their professional assessment [14,15].
- *Track changes over time*
  Bowel function and continence physiology change over the normal childhood developmental phases, which need to be reflected during assessment [16,17].
- *Validity*
  A validated assessment tool should ideally be used. Validation is conducted in each language the tool is used and should include culturally sensitive translation and a validity concept that refers to the comprehensibility and relevance of items [15,18].
- *Reliability*
  The tool should reflect the real bowel function, as experienced by the patients and their families. Reliability is given when the score produces the same or similar results in patients when repeatedly administered [14].
- *Assess continence with and without bowel management*
  Many children with HD require bowel management and may achieve social cleanness [8,16]. Bowel management approaches vary according to age and also clinician and family preference; hence, a variety of suppositories, enemas, rectal washouts, and transanal irrigation are used to relieve symptoms. The ideal bowel function assessment tool should incorporate the reality achieved with bowel management. It should also assess the degree of fecal incontinence by distinguishing between mild soiling and more severe fecal incontinence. It should differentiate between the different causes of soiling and/or fecal incontinence, such as critical reduction in sphincter tone, reflex incontinence due to an injured or removed anal canal, and outlet obstruction causing overflow incontinence (pseudo incontinence). Clinicians are interested in understanding both the real baseline bowel function to support and individualize the bowel management concept, with the ultimate aim of achieving continence or social continence in the latter case, as well as the achieved real-life continence [16,17]. It is of equal importance to assess the bowel function with bowel management as well. Thus, a score for patients with HD should provide insight into the patient’s bowel function and enable changes to be tracked with bowel management.
- *Specific groups/comorbidity*
  Common comorbidities in HD patients, such as trisomy 21, are associated with neurodevelopmental delay and other co-factors, e.g., muscular hypotonia, which may influence the ability to achieve continence as well.
  The criteria for bowel function assessment were established by the group of authors as the minimal requirement for a clinical score, following a review of existing scoring systems. The group reached full consensus.

### 2.3. OASIS Group Recommendation to Use the Rintala Bowel Function Score

The OASIS group recommends adopting the Rintala Bowel Function Score as the standard tool for assessing bowel function in HD. This score is widely recognized and consists of seven items that assess key domains such as defecation frequency, incontinence, constipation, and social problems. Its brevity and comprehensive nature make it a practical tool for clinical use. Importantly, it has been validated in Scandinavian countries and has been used extensively to evaluate both HD and anorectal malformation (ARM).

### 2.4. Real-Time Data Collection

The peculiarity of the assessment of intestinal function is characterized by the physiological changes with normal child development. A retrospective evaluation of electronic health records often lacks information, and the existing records are often subjective.

Using a standardized score may improve the comparability of the reported bowel function outcome. Combined with automated extraction from electronic records, monitoring may become easier. However, the information input relies on the meticulous dedication of a healthcare professional who is documenting a bowel function score [14]. 

Therefore, patient-reported outcome measures (PROMs) empower the patient and overcome the clinician factor. A semi-automated dashboard has the further potential of enabling automated data extraction from electronic health records [14].

## 3. Concluding Remarks

The OASIS group acknowledges the availability of a variety of bowel function scores, including both validated and non-validated scores. The participants agree that to compare results, a bowel function score should be used as a reference. The group points out that currently, the available literature on long-term outcomes is difficult to interpret due to the heterogeneity of the scores used. Clinicians reflect that it is challenging to perform bowel function assessments due to limited time during outpatient clinic appointments. Clinicians and patient representatives are also unsure if patients provide consistent answers when questioned by their surgeons.

Standardized bowel function questionnaires have been developed to provide a holistic view of patients’ bowel function and, at the same time, predict patient outcomes. Validated scores allow for objective comparison between patient groups and their longitudinal follow-up.

Considering the abundance of mostly self-designed scores used in pediatric colorectal disease, we question the need for a specific score for patients with Hirschsprung disease (HD). In the literature, patients with anorectal malformation (ARM) and HD are often pooled and discussed together [12], despite representing two distinct entities with disease-related specificities. A core set for the assessment of urinary and fecal incontinence was under development at the Zürich University of Applied Sciences, Switzerland, the ICF Incontinence Assessment Form (ICF-IAF), but it has not been completed yet. Unfortunately, it could not be brought to completion because of a lack of funding [19,20,21]. 

Children with HD, per pathophysiology, have an intact continence organ but may suffer from persistent outlet obstruction and overflow pseudo incontinence, which may present with soiling that may be misdiagnosed as incontinence [16]. Indeed, this outlet obstruction needs to be adequately addressed in the functional assessment and follow-up of patients with HD, ideally reflected in an HD-tailored continence score. However, it should be noted that surgical damage to the sphincters or squamous epithelium of the anal canal can also cause chronic fecal incontinence [4,16,22].

According to the OASIS-Holistic Care in Hirschsprung Disease Network Group, a bowel function score that can be used in patients with HD should quantify baseline variables, allow changes to be tracked over time, be valid and reliable, and assess continence with and without bowel management. To ensure compliance, the ideal score should include the above key features, but remain short and comprehensive.

Using PROMs may further help to understand outcomes that matter to our patients [22]. This is echoed by patient representatives at the OASIS-Holistic Care in Hirschsprung Disease Symposium, who stated that it is ultimately more important to patients that they experience a deviation from their normal peers than to be informed of their actual score.

Another general tool that can be used in follow-up to obtain a broad overview of bowel habits is a bowel diary [14]. By self-documenting daily bowel habits, including information on medications such as laxatives or stool softeners, as well as describing stool consistency according to the Bristol score, stool frequency, and the incidence of soiling or fecal accidents over a two-week period, the treating physician can obtain important information to guide treatment and also evaluate treatment effects in an objective way [15,23,24]. However, a bowel diary cannot be used as a tool to compare patient groups and can therefore only be used as a patient-centered diagnostic tool for care and follow-up. Longitudinal follow-up is important because bowel function can change throughout life [5].

Ideally, restorative surgery is performed in infancy when patients’ bowel function can be compared with that of healthy peers, and children are not yet physiologically toilet trained [1,2]. There is, of course, inter-individual variation in the timing of toilet training. In general, up to 53% of patients with HD have obstructive symptoms, up to 37% have Hirschsprung-associated enterocolitis (HAEC), and up to 45% have fecal incontinence symptoms that can change over time [3,5,22,25,26]. Monitoring bowel function over time allows the clinician to understand the evolution of bowel problems associated with HD and may also serve as a motivator for the patient and family [14]. As mentioned in the literature, it is commonly observed that the pathognomonic persistent outlet obstruction usually disappears once the patient reaches adolescence. Systematic monitoring using a consistent set of measurements can help to improve our understanding of bowel function in HD patients in relation to disease severity, surgical technique, and patient age, which will ultimately enable clinicians to manage and counsel their HD patients and parents in a disease-tailored manner in the future [5,6,27].

As there is no consensus among pediatric surgeons on scores to assess bowel function, a large number of different scores can be found in the literature. According to the aforementioned criteria for a bowel function assessment tool applicable to patients with HD, the bowel function score established by Rintala and Lindahl [28] is the most commonly used continence score. After an extensive discussion of the different scores described in the literature, the “Continence Score of the OASIS-Holistic Care in Hirschsprung Disease Network Group” considered Rintala and Lindahl’s bowel function score to be the most appropriate to assess bowel function in patients with HD. It is an established 7-item scoring system with domains of sensation and urge to defecate, ability to hold defecation, frequency of defecation, fecal continence, constipation, and social problems related to bowel function, with a maximum score of 20. It was originally developed for children with ARM and compared with an age-matched healthy population in Finland [28,29,30]. Since then, it has also been used worldwide to assess bowel function in patients with HD and has become the most popular and widely used bowel function score for HD in the last decade [3,5,10,27,31,32,33,34,35,36,37,38,39,40,41,42,43,44,45,46,47,48,49,50,51,52,53,54,55,56,57,58,59,60,61,62,63,64].

Scores have consistently correlated closely with clinical outcomes, and it is therefore considered a validated score in Scandinavian countries [28,33,35]. However, despite its worldwide use to assess bowel function in patients with ARM and HD, its degree of validation is not entirely clear, as cultural adaptation and validation concepts have not been applied elsewhere. The advantage of this questionnaire is that it is short, comprehensive, and observer-independent, as it can be completed by the child or their parents without the need for a clinical examination. 

Consistent use of a single score will ultimately lead to a reliable comparison of bowel function obtained from HD patient groups. In addition, consistent use of the same score will allow assessment of bowel function over time as individuals are followed, which will allow adaptation and optimization of treatment and thus contribute to the associated quality of life. Longitudinal monitoring is particularly important in patients with HD, as the bowel function is known to change during growth; up to 53% of HD patients experience obstructive symptoms despite corrective surgery, which has been described as a cause of Hirschsprung-associated enterocolitis (HAEC), which has been reported to occur in up to 37% of patients post-operatively, while 45% of patients even present with fecal incontinence [3,5,22,25,26]. Recent results have shown that bowel function does not necessarily improve over time and that adolescent and adult HD patients still have to cope with problems such as soiling, constipation, or even incontinence [6,65].

Therefore, targeted and evidence-based follow-up is paramount to assess bowel function and meet the long-term care needs of this patient cohort, which ultimately contributes to each patient’s quality of life.

Future developments will undoubtedly lead to PROMs that can be implemented in a structured long-term follow-up via apps where patients can report directly on their bowel function. This could be introduced as an automatic reminder, e.g., during the transition process. Self-reported outcome parameters have the advantage of being unbiased and reflecting the actual perception of the respondent. With the use of technology and artificial intelligence, this may be the optimal assessment tool to obtain a bowel function score in the future.

### Limitation

Our work contributes to the ongoing discussion on the most effective ways to evaluate and report outcomes in pediatric surgery, not only for assessing the current situation but also for gathering evidence by pooling patient data. However, this manuscript is limited to describing specific items and discussion points and reflects the collective opinion of the author group.

## 4. Key Message by OASIS-Holistic Care in Hirschsprung Group

The implementation of a standardized tool like the Bowel Function and Continence Scoring Questionnaire across clinicians treating patients with Hirschsprung disease (HD) is crucial for several reasons:

1. Consistency in Reporting: Uniform use of a single tool will lead to standardized reporting of bowel function outcomes, ensuring that patient data are comparable across different centers and clinical practices. This can enhance the quality of research and patient care, enabling the identification of trends and best practices.
2. Longitudinal Monitoring: Regular use of a scoring system will allow for continuous monitoring of each patient’s bowel function over time. Clinicians will be able to detect patterns of improvement, stabilization, or deterioration in symptoms as the patient grows, facilitating more personalized and proactive management strategies.
3. Integration with Patient-Reported Outcome Measures (PROMs): Incorporating the scoring tool alongside other PROMs enables clinicians to have a more holistic view of the patient’s experience. Patients and caregivers can contribute their perspectives on symptoms and quality of life, giving clinicians a fuller picture of the effectiveness of treatments.
4. Guiding Care and Follow-up: By consistently using these tools, clinicians will have objective data to guide decision-making in care pathways. Bowel function and continence scores can inform the need for interventions, adjustments in treatment plans, or further assessments, leading to more targeted and effective management.
  The focus on improving bowel function-related symptoms, especially as patients progress through growth milestones, will be essential for optimizing long-term outcomes in HD management.

## Figures and Tables

**Table 1:** Bowel function scores commonly used in Hirschsprung disease.

	Holschneider	Rintala	Wingspread	Krickenbeck	Baylor Continence Scale	Fecal Incontinence Index (FII)	Pediatric Incontinence and Constipation Score	Groningen Pediatric Defecation and Fecal Continence Questionnaire	Bowel Diary	ARM-Net
First used		1995	1986	2005	2007					
Filled out by whom	parents	parents		parents	patients	patients			parents	clinician
Validated					yes	yes Brazil				no
Number of items	7	7	4	3	23	8	13	75–88	4	
Includes type of ARM	no	no		no					no	
Includes type of Hirschsprung	no	no	no	no	no	no	no	no	no	
Incontinence	yes	yes	yes	yes	yes	yes		yes	Yes	yes
Constipation	yes	yes	yes	yes	yes	yes		yes	Yes	yes
Urinary problems	no	no	no		yes			yes	no	yes
Anal sensation	yes	no	no	no				yes	no	
Social problems	no	yes	no	no	yes			yes	no	
Includes results with bowel management			yes	yes				yes	yes	yes

## Data Availability

The original contributions presented in the study are included in the article, further inquiries can be directed to the corresponding author.

## Abbreviations

ARM	Anorectal malformation
ERNICA	European Reference Network for rare Inherited and Congenital (digestive and gastrointestinal) Anomalies
HAEC	Hirschsprung-associated enterocolitis
HD	Hirschsprung disease
ICF	International Classification of Functioning, Disability and Health
PROM	Patient-reported outcome measures
QoL	Quality of life
